# Endocrinopathies in Inborn Errors of Immunity

**DOI:** 10.3389/fimmu.2021.786241

**Published:** 2021-11-23

**Authors:** Kei Takasawa, Hirokazu Kanegane, Kenichi Kashimada, Tomohiro Morio

**Affiliations:** ^1^ Department of Pediatrics and Developmental Biology, Tokyo Medical and Dental University (TMDU), Tokyo, Japan; ^2^ Deparment of Child Health Development, Tokyo Medical and Dental University (TMDU), Tokyo, Japan

**Keywords:** endocrinopathy, thyroiditis, diabetes mellitus, growth failure, inborn errors of immunity (IEIs), HCT

## Abstract

Inborn errors of immunity (IEI), caused by hereditary or genetic defects, are a group of more than 400 disorders, in which the immune system, including lymphocytes, neutrophils, macrophages, and complements, does not function properly. The endocrine system is frequently affected by IEI as an associated clinical feature and a complex network of glands which regulate many important body functions, including growth, reproduction, homeostasis, and energy regulation. Most endocrine disorders associated with IEI are hypofunction which would be treated with supplementation therapy, and early diagnosis and appropriate management are essential for favorable long-term outcomes in patients with IEI. In this review, we aimed to comprehensively summarize and discuss the current understanding on the clinical features and the pathophysiology of endocrine disorders in IEI. This review is composed with three parts. First, we discuss the two major pathophysiology of endocrinopathy in IEI, autoimmune response and direct effects of the responsible genes. Next, the details of each endocrinopathy, such as growth failure, hypothyroidism, hypoparathyroidism, adrenal insufficiency, diabetes mellitus (DM) are specified. We also illustrated potential endocrinopathy due to hematopoietic stem cell transplantation, including hypogonadism and adrenal insufficiency due to glucocorticoid therapy.

## Introduction

Inborn errors of immunity (IEI), caused by hereditary or genetic defects, are a group of more than 400 disorders, in which the immune system, including lymphocytes, neutrophils, macrophages, and complements, does not function properly (1–4). While the clinical characteristics of each IEI differs, defects in the functions of the immune system generally lead to increased susceptibility to infection, which can be life-threatening or can cause permanent damage to various organs. Although the clinical symptoms of IEI are generally present at birth or in early childhood, patients can be affected with IEI at any age (1–4).

In addition to opportunistic infections, various clinical features are also involved in IEI. Autoimmunity and abnormal inflammation in the absence of apparent infection have often been observed clinically in association with IEI, and IEI has been linked to specific autoimmune/allergic complications at various frequencies (5). Furthermore, numerous causative genes of IEI are involved in the fundamental functions of cell differentiation and proliferation and are shared by various types of cells. Taken together, the clinical problems caused by the impaired molecular function of IEI genes are not limited to immunological systems (5).

Clinical complications of IEI can be classified into three groups according to the causes, the direct organ damages from opportunistic infections, chronic autoimmune/allergic inflammation, and impaired molecular functions of the causative genes (2, 5). Further, abnormal cell proliferation or carcinogenesis due to IEI could be form the pathogenesis of endocrinopathy. In any case, every organ can be affected by IEI, and for the clinical management of IEI, interventions for both opportunistic infections and clinical complications are essential.

The endocrine system is not an exception and is frequently affected by IEI as an associated clinical feature (**Table 1**) (12). The endocrine system is a complex network of glands that produce and release hormones which regulate many important body functions, including growth, reproduction, homeostasis, and energy regulation. During childhood, the endocrine system is essential for acquiring normal growth and secondary sexual characteristics (13), and pediatric endocrinopathy seriously affects long-term outcomes. On the other hand, most endocrine disorders associated with IEI are hypofunctions that can be treated with supplementation therapy. Therefore, early diagnosis and appropriate management are essential for favorable long-term outcomes in patients with IEI. In this review, we aim to summarize and discuss the current understanding of the clinical features and pathophysiology of endocrine disorders in IEI.

**Table 1:** Modified the classification of the International Union of Immunological Societies Expert Committee according to complications of endocrinopathy (2).

Category		Disease	Gene	Inheritance	OMIM	Endocrinopathies							
						growth failure	thyroiditis	diabetes mellitus	adrenal insufficiency	hypoparathytoidism	hypogonadism	dyslipidemia	
Immunodeficiencies affecting cellular and humoral immunity	T-B- SCID	Artemis deficiency	DCLRE1C	AR	605988	X							
		Ligase 4 deficiency	LIG4	AR	601837	X							
	Combined immunodeficiency (CID), generally less profound than SCID	Polymerase and deficiency	POLD1, POLD2	AR	174761, 600815	X							
Combined immunodeficiencies with associated or syndromic features	DNA repair defects	Ataxia-telangiectasia	ATM	AR	607585			X			X		*
		Bloom syndrome	BLM	AR	604610	X		X					
		Ligase I deficiency	LIG1	AR	126391	X							
		MCM4 deficienc	MCM4	AR	602638	X			X				
		POLE1 (Polymerase ϵ subunit 1) deficiency (FILS syndrome)	POLE1	AR	174762	X							
		POLE2 (Polymerase ϵ subunit 2) deficiency	POLE2	AR	602670		X	X					
		RNF168 deficiency (Radiosensitivity, Immune Deficiency, Dysmorphic features, Learning difficulties [RIDDLE] syndrome)	RNF168	AR	612688	X							
	Thymic defects with additional congenital anomalies	Chromosome 11q deletion syndrome (Jacobsen syndrome)	11q23del	AD	147791	X							
		Chromosome 10p13-p14 deletion syndrome (10p13-p14DS)	Del10p13-p14	AD	601362	X				X			
		DiGeorge/velocardio-facial syndrome Chromosome 22q11.2 deletion syndrome (22q11.2DS)	Large deletion (3 Mb) typically in chromo- some 22 (TBX1)	AD	602054					X			
		CHARGE syndrome	CHD7	AD	214800	X					X		**
	Immuno-osseous dysplasias	Immunoskeletal dysplasia with neurodevelopmental abnormalities (EXTL3 deficiency)	EXTL3	AR	617425	X							
		MYSM1 deficiency	MYSM1	AR	612176	X							
		MOPD1 deficiency (Roifman syndrome)	RNU4ATAC	AR	601428	X							
		Schimke immuno-osseous dysplasia	SMARCAL1	AR	606622	X							
	Hyper IgE syndromes (HIES)	PGM3 deficiency	PGM3	AR	172100	X							
	Other defects	KMT2A deficiency (Wiedemann-Steiner syndrome)	KMT2A	AD	605130	X							
		Kabuki syndrome (type 1 and 2)	KMT2D KDM6A	AD XL	602113 300128	X							
		Activating de novo mutations in nuclear factor, erythroid 2- like (NFE2L2)	NFE2L2	AD	617744	X							
		STAT5b deficiency	STAT5B	AR	245590	X							
		STAT5b deficiency	STAT5B dominant negative	AD	604260	X							
Predominantly antibody deficiencies	Severe reduction in all serum immunoglobulin isotypes with profoundly decreased or absent B cells, agammaglobulinemia	X-linked agammaglobulinemia (XLA)	BTK	XL	300300	X							
		NFKB1 deficiency	NFKB1	AD	164011		X						
		NFKB2 deficiency	NFKB2	AD	615577				X				***
		Activated p110δ syndrome (APDS) type2	PIK3R1	AD	616005			X					†
Diseases of immune dysregulation	Regulatory T cell defects	IPEX, immune dysregulation, polyendocrinopathy, enteropathy X-linked	FOXP3	XL	300292		X	X					
		CTLA4 Haploinsufficiency with autoimmune infiltation	CTLA4	AD	616100		X	X	X				††
		STAT3 GOF mutation	STAT3	AD	102582		X	X					
		LRBA deficiency	LRBA	AR	614700		X	X					
	Autoimmunity with or without lymphoproliferation	APECED (APS-1), autoimmune polyendocrinopathy with candidiasis and ectodermal dystrophy	AIRE	AR or AD	240300	X		X	X	X	X		
		ITCH deficiency	ITCH	AR	606409		X	X					
		JAK1 GOF	JAK1	AD GOF	147795	X	X						
Congenital defects of phagocyte number or function	Congenital neutropenias	Glycogen storage disease type 1b	G6PT1	AR	602671							X	
		Kostmann Disease, SCN3	HAX1	AR	605998						X		†††
		JAGN1 deficiency	JAGN1	AR	616012								
		P14/LAMTOR2 deficiency	LAMTOR2	AR	610389	X							
		Barth Syndrome (3-Methylglutaconic aciduria type II)	TAZ	XL	300394	X							
	Defects of motility	β actin deficiency	ACTB	AD	102630	X							
		Leukocyte adhesion deficiency type 2 (LAD2)	SLC35C1	AR	605881	X							
Defects in intrinsic and innate immunity	Predisposition to mucocutaneous candidiasis	mucocutaneous candidiasis	STAT1 GOF	AD GOF	600555		X	X					
	Type 1 interferonopathies	Congenital Osteopetrosis	TNFSF11	AR	602642	X							
Autoinflammatory disorders	Type 1 interferonopathies	Spondyloenchondro-dysplasia with immune dysregulation (SPENCD)	ACP5	AR	171640	X							
Bone marrow failure		Bone marrow failure	DKCB5	AR	615190	X							
		Fanconi Anemia, TypeA, B, C, D1, D2, E, F, G, I, J, L, M, N, O, P, Q, R, S, T, U, V, W	FANCA FANCB FANCC BRCA2 FANCD2 FANCE FANCF XRCC9 FANCI BRIP1 FANCL FANCM PALB2 RAD51C SLX4 ERCC4 RAD51 BRCA1 UBE2T XRCC2 MAD2L2 RFWD3	AR or XLR	227650 300514 227645 605724 227646 600901 603467 614082 609053 609054 614083 618096 610832 613390 613951 615272 617244 617883 616435 617247 617243 617784								
		MIRAGE (myelodysplasia, infection, restriction of growth, adrenal hypoplasia, genital phenotypes, enteropathy)	SAMD9	AD GOF	617053				X		X		
		Coats plus syndrome	STN1, CTC1	AR, AR	613129, 617053								

References of additional information are indicated as follows.

* (6): ** (7): *** (8).

^†^ (9): ^††^(10): ^†††^(11).

## Pathophysiology of Endocrinopathy in IEI

### Autoimmune

The immune system becomes self-tolerant through mechanisms called “tolerance”. Induction of tolerance is accomplished by education of both B and T cells, which occurs in both central (bone marrow and thymus) and peripheral (spleen and lymph nodes) lymphoid organs, and immune dysregulation causes autoimmune/allergic responses (14–17). Autoimmune diseases can be classified into systemic diseases, such as systemic lupus erythematosus, rheumatoid arthritis (RA), and systemic sclerosis, and organ-specific diseases, such as type 1 diabetes mellitus (T1DM) and autoimmune thyroiditis (17). Endocrine organs are the major targets of organ-specific autoimmune diseases, and most endocrinopathies in IEI are presumably mediated by autoimmunity (14–16).

The most common autoimmune endocrine disorders are autoimmune thyroid disorders and T1DM (2). Despite extremely rare conditions, hypophysitis, adrenalitis, ovarian failure, and hypoparathyroidism can occur due to an autoimmune response. Autoimmune endocrine disorders are characterized by associations with autoantibodies and/or autoreactive lymphocytes, which result from an interaction between environmental factors and genetic predisposition. For a genetic contribution to autoimmune endocrinopathies, epidemic studies of twins provide robust evidence that monozygotic twins have a higher concordance rate for disease than dizygotic twins, and familial accumulation is frequently observed. Most autoimmune endocrine disorders have been reported to be associated with specific major histocompatibility complex (MHC)/human leukocyte antigen (HLA) molecules.

Despite the long history of intensive investigations, the precise genetic mechanisms underlying autoimmune endocrine disorders are poorly understood. One of the possible explanations is the “autoimmune surveillance of hypersecreting mutants” (ASHM) hypothesis. The presence of autoreactive T cells whose nature is detecting self-antigens from hormone secretion pathway, dedicates to detect and to remove malfunctioned endocrine cells which potentially disrupt organismal homeostasis. The speculation looks persuasive, nevertheless, the speculation does not clarify the reason why certain endocrine organs, such as thyroid gland and beta cells, tend to be more affected.

A recent significant expansion of our understanding of monogenic IEI, which is associated with autoimmune endocrine disorders, would provide valuable insights into their pathophysiology.

**Figure 1:**
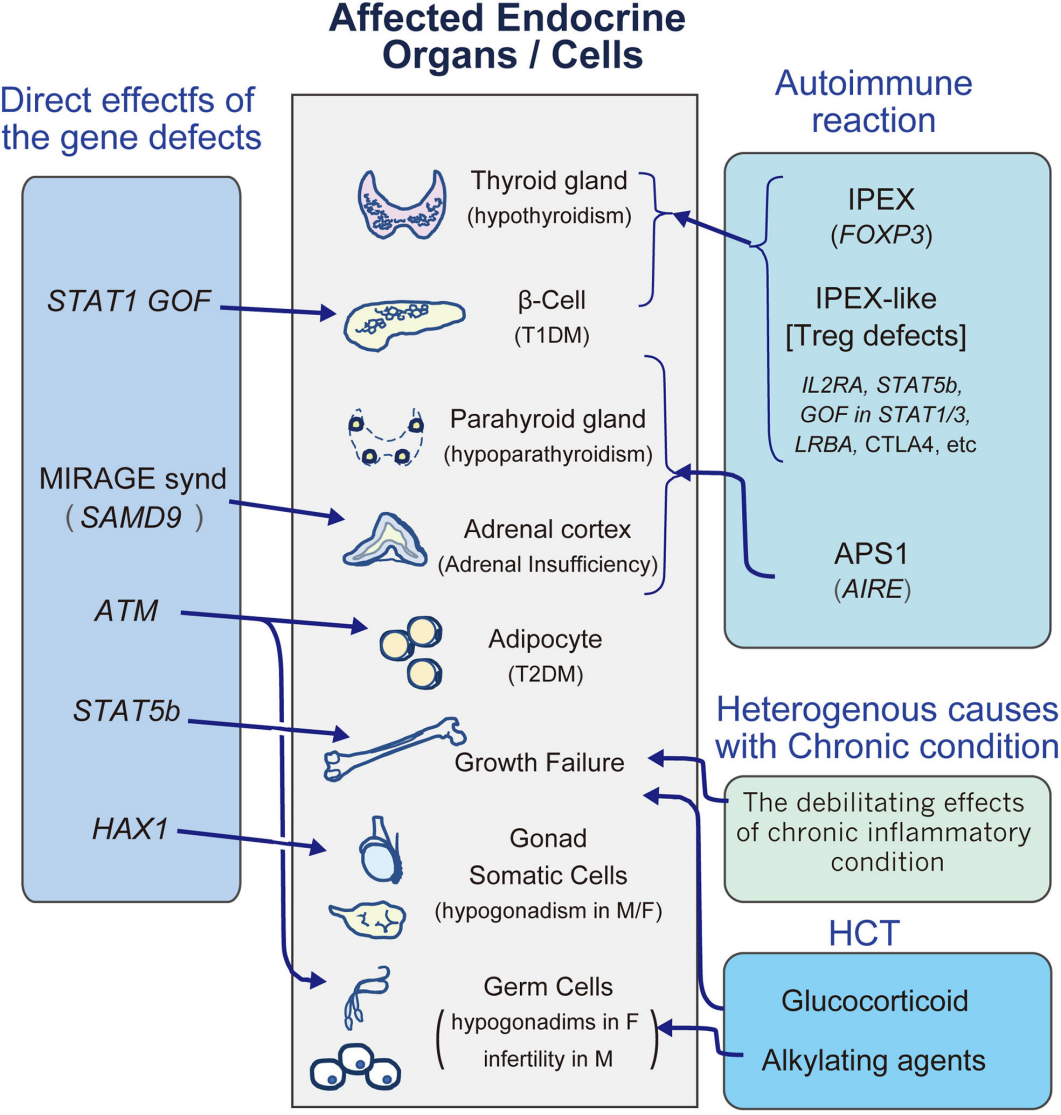
A diagram to review the major endocrinopathies in IEI. M, male; F, female; HCT, hematopoietic stem cell transplantation; T1DM, type1 diabetes mellitus; T2DM, type2 diabetes mellitus.

Classically, autoimmune polyendocrinopathy candidiasis ectodermal dystrophy (APECED) and immunodeficiency, polyendocrinopathy, and enteropathy X-Linked syndrome (IPEX) exemplify monogenic autoimmune disorders associated with endocrine disorders (**Figure 1**). From the discovery of the *AIRE* gene in APECED (18, 19), defects in central tolerance with alteration of self-antigen expression levels in the thymus would be a cause of T1DM, and strictly controlled negative selection of T cells through tissue-specific self-antigen expression in the thymus is essential for preventing autoimmune endocrine disorders (20). Studies of another IEI associated with T1DM, IPEX syndrome have shown that the responsible gene, *FOXP3* (21, 22), plays a critical role in the function of CD4^+^CD25^+^ regulatory T (Treg) cells (23, 24), and its defects cause loss of peripheral Tregs, leading to autoreactive T cell activation and proliferation (20, 23, 24).

Recent studies have revealed that other molecules are involved in monogenic autoimmune disorders involving endocrinopathies. In cohorts of subjects with an IPEX-like phenotype, up to half do not exhibit pathogenic variant in *FOXP3*, and recent investigations revealed that a number of gene defects involved in Treg cell function cause an IPEX-like phenotype, such as IL2RA, STAT5b, GOF in STAT1/3, and LRBA (**Figure 1**) (25–32).

Additionally, several monogenic disorders in immune function have also been reported to be involved in T1DM. Immune checkpoint proteins play a central role in balancing effective immune responses to pathogens, as well as regulating autoimmune responses against self-tissues. CTLA4 is one of the components, and heterozygous deficiency of the protein gives rise to inappropriate polyclonal T-cell activation (10, 33, 34), leading to highly variable features of autoimmune responses, which may include endocrinopathies of adrenal insufficiency, T1DM, and thyroiditis (35). On the other hand, anti-CTLA-4 antibody therapy, which is a novel therapeutic approach to malignancy by targeting immune checkpoint proteins, is associated with serious immune-related adverse events, such as autoimmune hypophysitis (36, 37).

Endocrinopathy would be comorbidity of recently identified IEI. Inherited PD-1 deficiency and activated PI3Kδ syndrome (APDS) type 2, would associate with T1DM (38–40). Familial thyroiditis and T1DM are reported in patients with A20 (TNFAIP3) haploinsufficiency (41, 42).

Autoimmune endocrine disorders are progressive and are considered untreatable and irreversible. Generally, the treatment of endocrinopathy involves hormone supplementation instead of immunological therapy. However, the experience of endocrine disorders in IEI would provide valuable insights for developing novel therapeutic approaches for autoimmune endocrine disorders. Early hematopoietic cell transplantation (HCT) for IPEX appears to reverse the autoimmune complications, including T1DM, suggesting that Treg cells may be more tractable for the induction of tolerance and could be a possible target for the radical treatment of T1DM (9, 17, 43). Consistently, administration of ruxolitinib, an inhibitor of Janus kinase (JAK) 1 and 2 reversed hyperglycemia of T1DM in a case of STAT1 GOF (44).

### Direct Effects of the Responsible Genes

Some endocrine disorders in IEI are thought to be caused by the direct influence of molecular defects (**Figure 1**).

STAT5b is a transcription factor that activates the transcription of *IL-2Rα, FOXP3*, and *Bcl-2*, and its deficiency exhibits combined immunodeficiencies with a reduced number of Treg cells and increased IgE (27, 45). The JAK2 and STAT pathways (STAT1, STAT3, STAT5A, and STAT5B) are also involved in the growth hormone (GH) receptor pathway, and we will discuss the details in C-2.

Kostmann disease is a type of severe congenital neutropenia (SCN) caused by *HAX1* deficiency (46). HAX-1 is involved in the regulation of apoptosis and plays a role in myeloid differentiation. Cekic et al. investigated seven female patients with severe neutropenia due to the p.Trp44X variant in the *HAX1* gene, and all female patients exhibited ovarian insufficiency. *HAX1* mRNA is abundantly expressed in the testes and ovaries, and the authors speculated that the *HAX1* gene plays an important role in ovarian development (11).

MIRAGE syndrome is a rare condition characterized by myelodysplasia, recurrent infection, restriction of growth, adrenal hypoplasia, genital phenotypes, and enteropathy. It is an autosomal dominant disorder typically caused by a *de novo* pathogenic variant of *SAMD9/SAMD9L*, whose gain of function variant increases the role of the molecule as a negative regulator of cellular proliferation by altering the endosome system (47, 48). This results in cytopenia, bone marrow failure, and immunodeficiency. In patients with MIRAGE syndrome, the adrenal glands are hypoplastic with severely disorganized structures, resulting in adrenal insufficiency. These findings are distinct from those of Addison’s disease caused by autoimmune inflammation (47–50).

*ATM* (ataxia telangiectasia, mutated) is essential for DNA repair, and its defects lead to ataxia telangiectasia (AT) with multisystem syndrome, including combined immunodeficiencies (51). Diabetes mellitus (DM) with severe insulin resistance is a major complication of AT. A study of AT mice model revealed that ATM regulates adipocyte differentiation, and in AT patients, adipocyte differentiation is disturbed, resembling lipodystrophy, which is one of the major causes of insulin resistance (52). *ATM* is also essential for germ cell meiosis. In mice, *Atm* deficiency results in severe meiotic disruption as early as leptonema of prophase I (53), resulting in germ cell depletion. Consistently, hypogonadism in AT is common only in female patients (6), because in contrast to testes, ovarian development is germ cell dependent.

*STAT1* gain-of-function (GOF) variants impair the development of IL-17-producing T cells, resulting in mucocutaneous candidiasis (28, 31). It also causes autoimmune endocrinopathies, such as thyroiditis and T1DM. In a meta-analysis, autoimmune thyroiditis and T1DM were observed in 23.0% and 5% of *STAT1* GOF patients, respectively (54). On the other hand, recent *in vitro* and *in vivo* studies have demonstrated an essential role for STAT1 in islet cell death. *Stat1* ablation in T1DM model mice (NOD mice) ameliorated diabetes and insulitis, suggesting that in addition to autoimmune mechanisms, GOF of *STAT1* would directly contribute to the pathogenesis of diabetes by inducing apoptosis of the islet beta cells (55).

CAHRGE syndrome is a genetic syndrome with known pattern of features, coloboma, heart anomaly, choanal atresia, intellectual disability, genital and ear anomalies (56, 57). Heterozygous *CHD7* gene deficiency causes most cases, and CHD7 protein regulates gene expression by chromatin remodeling (58). Immunodeficiency in CHARGE syndrome is rare, and occurs largely due to impairment in thymic development, and the severity of the immunodeficiency relates to the degree of thymic maldevelopment (7). The severest cases could be complete/near complete absence of T-cells and abnormal B-cell function with hypo-gammaglobulinemia. The syndrome is associated with impaired development of the pharyngeal arch structures, and in this regard, the clinical phenotypes overlap with 22q11.2 deletion syndrome (7). In addition to growth retardation, gonadotropin deficiency is one of the major clinical features of CHARGE syndrome. It causes micro penis in male and absent of pubertal development in both sexes (59).

## Endocrine Disorders Associated With IEI

### Multiorgan Endocrinopathy

#### APECED (APS-1)

APECED, also called autoimmune polyglandular syndrome type 1 (APS-1), is a rare condition with an approximate prevalence of 1:100,000 in US (60). and recessively inherited disorder caused by variants of the autoimmune regulator gene, *AIRE*. In addition to chronic mucocutaneous candidiasis (CMC) which is caused by the reduction of key cytokines for CMC, IL-22 and IL-17F (61), APECED is characterized by variable combinations of endocrine autoimmune diseases (62). *AIRE* consists of 14 exons spanning 11.9 kb of genomic DNA and encodes a 545-amino acid protein with a molecular weight of 58 kDa. *AIRE* plays an essential role in central tolerance induction by controlling the expression of tissue-specific self-antigens within the thymus. Disruption of *AIRE* function results in spontaneous autoimmunity in multiple organs with autoantibodies against organ specific antigens, such as TPO, Tg (thyroid gland), calcium sensing receptor (parathyroid gland), steroidogenic enzyme (adrenal cortex and gonad) and GAD/IA2/insulin (islet cells) (60).

In a classic large cohort study of 68 patients, the most common endocrinopathies were hypoparathyroidism and adrenal insufficiency (Addison’s disease), both of which were observed in more than 70% of patients (63). The onset of the two conditions occurs from early childhood and can be obvious at any age. Ovary is also would be a major target of autoimmune response, and approximately 40–70% of women with APECED will develop premature ovarian failure (63–65). On the other hand, T1DM and hypothyroidism affects less than 5-10% and 20-25% of the patients, respectively (65), and the age of onset is during adulthood than childhood.

In addition to endocrinopathy, all patients exhibit oral candidiasis and other problems, such as alopecia, vitiligo, and keratopathy, which affect the patients at various frequencies, and one of the possible causes is related to autoimmunity to interleukin (IL)-17 cytokines (66).

#### IPEX/IPEX Like Syndrome

IPEX syndrome is caused by loss of function of X linked gene, *FOXP3* and characterized by immunodysregulation, polyendocrinopathy, enteropathy. The pathophysiology of IPEX syndrome is explained by defects for the development, survival, and/or function of Treg cells. In addition to “classic IPEX”, a group of patients with an IPEX- like phenotype without pathological variant in *FOXP3* gene emerged, and several genes, such as *IL2RA, STAT5b, GOF* in *STAT1/3, LRBA* and *CTLA4*, have been identified as causative genes for IPEX-like phenotype. Recently, for categorizing the group of IPEX and IPEX like diseases, in which Treg deficiency caused by monogenic deficiency, “Tregopathies” is proposed (67).

Among the IPEX and IPEX- like patients, most common endocrinopathies are T1DM and thyroiditis. Additionally, few patients, less than 5%, develop adrenal insufficiency. In a cohort of 173 IPEX/IPEX like patients, endocrinopathy were seen at a similar frequency (IPEX 65% vs. IPEX-like 59%). On the other hand, there was almost a two-fold difference in the frequency of patients with T1DM in the IPEX cohort compared to the IPEX-like group (IPEX 49% vs. IPEX-like 28%) (32). Clinical thyroid disease was more prevalent in the IPEX-like group (IPEX 26% vs. IPEX-like 39%), and approximately more than 70% of cases with thyroiditis are associated with T1DM (68). Of IPEX like subjects, *STAT5b* and *STAT1/3* GOF more likely evolve endocrinopathy with more than 90% of frequency (32).

### Growth Failure

Endocrinologically, GH and thyroid hormone mainly contribute to linear growth, and deficiencies in these hormones lead to growth failure. However, other causes affect linear growth, e.g., undernutrition, rheumatologic inflammation, chronic renal failure, malignancy, respiratory failure, severe cardiac diseases, and genetic disease with primary effects on growth. Indeed, despite the risk of growth failure in IEI (69), GH deficiency or hypothyroidism is not documented in most IEI patients with growth failure (70). This suggests that the causes of growth failure in patients with IEI are heterogeneous.

Furthermore, glucocorticoids are strong growth suppressors, and the treatments for IEI would also cause growth failure. Few studies have evaluated treatments that affect linear growth in IEI. A retrospective study suggested that after HCT for IEI, linear growth was generally decreased rather than increased, and the glucocorticoid treatment duration for chronic graft-versus-host disease (cGVHD) was an independent risk factor for growth inhibition (71).

Among the causative genes for IEIome genes are directly involved in linear growth. The JAK-STAT signaling pathway plays an essential role in the immune system, and homozygous disruption of one of the components, STAT5b, leads to hyperimmunoglobulin E (IgE) syndrome (HIES) with low NK-cell numbers and modest T cell lymphopenia. STAT signals are also located downstream of the GH receptor (72), and a homozygous variant in *STAT5b* causes growth failure with GH insensitivity, i.e., normal serum GH levels with very low IGF-1 levels (45). Short stature in STAT5b defects is severe [-9.9 to -4.3SD), and GH therapy will not ameliorate growth failure. Although there are few data, IGF-1 therapy would be an option for short stature homozygous or heterozygous variants in *STAT5b* (73).

Although STAT3 is also involved in the GH signaling pathway, normal growth is maintained in the heterozygous loss of function of *STAT3* in HIES because STAT3 may negatively regulate GH-induced STAT5b (74, 75). Indeed, the GOF variant of STAT3 inhibits linear growth in several hematologic autoimmune disorders (75).

### Thyroiditis

The thyroid gland is a butterfly-shaped organ located at the base of the neck. It releases the thyroid hormones, T3 and T4, and its serum levels are strictly regulated by the TRH–TSH–thyroid axis without apparent circadian rhythm. Thyroid hormones regulate numerous body functions by controlling metabolism. During infancy and childhood, thyroid hormones also play essential roles in growth and brain development. Therefore, diagnosis and interventions for thyroid hypofunction (hypothyroidism) during childhood should not be delayed (76).

Except for congenital hypothyroidism and iodine deficiency, most cases of hypothyroidism are caused by autoimmune thyroiditis. Autoimmune thyroiditis is one of the most common endocrinopathies, with an estimated prevalence of 350 cases/100,000/year (95% confidence interval [CI]: 280–450) in women and 60 cases/100,000/year (95% CI: 30–120) in men (77). Most patients with thyroiditis exhibit no other immunological problems, such as systemic autoimmune diseases or immunodeficiencies (78). In contrast, patients with any problems in the immune system are at a high risk for thyroiditis. IEI is not an exception, and to date, a substantial number of IEI are reported to be associated with thyroiditis (17).

The clinical symptoms and signs of hypothyroidism are insidious and non-specific. Inactivity, general fatigue, and emotional symptoms linked to depression are major symptoms of hypothyroidism in adults (78). Except for growth retardation and developmental delay, clinical symptoms are reversible by LT4 supplementation therapy, and patients at high risk for hypothyroidism should be closely monitored. Regular examination of thyroid function (TSH, fT4, and fT3) is the most efficient approach (78). Frequent evaluation of neurological development and tracking of growth with a growth chart are essential for monitoring high-risk children. Autoantibodies for thyroid glands, such as TPO-Ab and Tg-Ab, would be excellent biomarkers for autoimmune thyroiditis, and euthyroid patients with TPO-Ab and/or Tg-Ab should be carefully followed up with regularly repeated thyroid function because of the risks for evolving hypothyroidism (77, 79).

Autoimmune thyroiditis occasionally cause hyperthyroidism, namely Graves’ disease. In this condition, autoantibodies that bind to the thyrotropin receptor (TSHR-Ab) stimulate thyroid gland in a TSH independent manner, leading to overproduction of thyroid hormone (80). The backgrounds of autoimmunity between hypothyroidism and hyperthyroidism are identical, and the patients with autoimmune thyroiditis are at high risk for developing hyperthyroidism. Many studies simply noted “thyroiditis”, which may evolve into either hyperthyroidism or hypothyroidism, and the epidemiological details of Graves’ diseases in IEI are not precisely clarified. The diagnosis of Graves’ disease can be confirmed by undetectable levels of TSH (< 0.3 mU/l), high serum free T4 and T3 levels, and the presence of TSH receptor antibodies (TRAb) (80). To date, associations with selective IgA deficiency, IPEX and Wiskott–Aldrich syndrome are reported (81–86). The clinical symptoms of hyperthyroidism are insidious as hypothyroidism, and regular examination of thyroid function for high risk group are essential.

### Hypoparathyroidism

The parathyroid glands produce PTH and maintain serum calcium levels. The major function of PTH is to increase bone absorption, inhibit calcium excretion from the kidney, and activate 1αhydroxylase, which synthesizes active vitamin D3, 1-25(OH)_2_ Vit D3 (87). The serum calcium level is strictly regulated by PTH because of its essential role in numerous biological functions. When PTH secretion is insufficient as in hypoparathyroidism, hypocalcemia develops, which can cause various clinical problems, including tetany, arrhythmia, prolonged QT interval, hypotension, and neurological complications, such as seizures (87). Aside from postsurgical hypoparathyroidism, the major causes of hypoparathyroidism include abnormal parathyroid development and autoimmune-mediated destruction (87).

22q11.2 deletion syndrome (also known as DiGeorge syndrome) is the most common microdeletion syndrome in humans, with an incidence of 1:4000 live births. Its clinical manifestations are highly variable, including congenital heart disease, palatal abnormalities, immune deficiency due to thymic hypoplasia with fewer T cells, characteristic facial features, hearing impairment, and hypoparathyroidism. The cause of hypoparathyroidism in 22q11.2 deletion syndrome is congenital defects, and the onset of hypocalcemia occurs mainly during the neonatal period. However, recent analysis revealed that autoimmune responses are involved in the pathogenesis of hypoparathyroidism, and hypocalcemia may occur at any age (88). These findings suggest that routine screening of serum calcium levels is essential for the long-term follow-up of the syndrome.

Chromosome 10p13-p14 deletion syndrome (10p13-p14DS) was originally discovered in patients with clinical phenotypes resembling 22q11.2 deletion syndrome. The syndrome also has a wide variety of clinical symptoms, including immunodeficiency and hypoparathyroidism (89, 90). Although the etiology of hypoparathyroidism in 10p13-p14 deletion syndrome is thought to be congenital defects, the involvement of autoimmune responses can be considered as 22q11.2 deletion syndrome. Further accumulation of cases with long-term follow-up of hypoparathyroidism would provide valuable insights for hypoparathyroidism mediated by autoimmune mechanisms in 10p13-p14 deletion syndrome.

The clinical features of APECED including hypopara-thyroidism are discussed in C-1.

### Adrenal Insufficiency

Adrenal insufficiency is a disorder that occurs when the adrenal glands are not capable of producing sufficient adrenal hormones, mainly cortisol. Cortisol protects the body from physical stress and is essential for life. Therefore, adrenal insufficiency can be life-threatening, requiring prompt and appropriate diagnosis. However, the diagnosis of adrenal insufficiency is challenging. First, the common symptoms are non-specific, such as fatigue, muscle weakness, loss of appetite, weight loss, and abdominal pain. Second, one-spot blood sampling is usually insufficient for the endocrinological diagnosis, and precise studies, including ACTH stimulation test or circadian rhythm, are required. Thus, identifying patients who are at a high risk for adrenal insufficiency is crucial (91).

The production of cortisol is strictly regulated by the hypothalamus–pituitary–adrenal (HPA) axis. Except for iatrogenic adrenal insufficiency due to glucocorticoid therapy, major causes of adrenal insufficiency are deficiency of ACTH secretion from the pituitary gland (secondary adrenal insufficiency) and organ failure of the adrenal gland (primary adrenal insufficiency) (91, 92).

Most causes of ACTH deficiency are anatomical defects of the pituitary gland, in which the production of multiple pituitary hormones is impaired. Thus, isolated ACTH deficiency is rare (93). Recently, loss-of-function variants of *NFKB2* have been shown to cause adrenal insufficiency due to isolated ACTH deficiency (8, 94, 95).

NFKB2 belongs to the NF-kB family, which is a group of evolutionarily conserved transcription factors involved in organ development, immune systems, and oncogenesis. Impaired function of NF-kB transcription factors or their regulators has been reported to be associated with primary immunodeficiency and autoimmunity (96). Classically, the condition characterized by a combination of common variable immunodeficiency (CVID) and ACTH insufficiency is called DAVID syndrome. ACTH deficiency occurred in 44% of pathologic variant carriers with a median age at onset of 8.2 years (SD ± 4.2, median 7.0, range 4–15 years) and is difficult to predict based on genetic alterations. The age at onset of ACTH deficiency suggests that the condition is acquired rather than congenital, and ACTH deficiency is presumed to be caused by T cell-mediated autoimmune diseases (8).

Except for congenital conditions, primary adrenal insufficiency is mainly caused by an autoimmune response. Isolated adrenal insufficiency accounts for up to 40% of patients with autoimmune primary adrenal insufficiency, and one or more organ-specific autoimmune endocrinopathies, such as autoimmune thyroid disease, T1DM, and premature ovarian insufficiency, can be associated with adrenal insufficiency. 21-Hydroxylase is a major autoantigen in autoimmune primary adrenal insufficiency (97), and the etiologies of primary adrenal insufficiency can be screened for using 21-hydroxylase antibodies (98). Of numerous genes that cause autoimmune adrenal insufficiency, *AIRE* is a representative gene that is associated with IEI (92). A recent cohort study of 691 patients with autoimmune adrenal insufficiency reported that variants of the *CTLA4* gene would increase susceptibility to adrenal insufficiency (99).

Recently another IEI gene has been identified to cause primary adrenal insufficiency. MCM4 (mini chromosome maintenance deficient 4 homolog) is part of a hetero-hexameric helicase complex which is important for DNA replication and genome integrity, and pathological variations were identified in an Irish travelling community manifesting with familial adrenal insufficiency (100, 101). Adrenal insufficiency is progressive and onset was usually in childhood following a period of normal adrenal function. ACTH resistance without mineralocorticoid deficiency is the characteristic of the condition. Although NK cell function is disrupted in the syndrome, the precise mechanisms of adrenal insufficiency are remained to be clarified (102).

Adrenal hypoplasia due to *SAMD9* is discussed in B-2.

### Diabetes Mellitus

DM is a condition of chronic hyperglycemia due to the dysregulation of glucose homeostasis. Insulin is the only hormone that decreases blood sugar, and the pathophysiology of diabetes can be classified into two main groups: insulin deficiency and increased insulin resistance. The major pathogenesis of insulin deficiency is an autoimmune response. A targeted immune response by both T and B cells leads to the destruction of insulin-producing β-cells in the islets of the pancreas. This condition is called T1DM, and some IEI are frequently associated with T1DM, such as APS type 1 (*AIRE*), IPEX (*FOXP3*), *CTLA4*, *LRBA*, and GOF of *STAT1* (22, 31, 35, 63, 103).

One distinctive clinical feature of T1DM associated with IEI is age at onset. Generally, an average age of onset of childhood-onset T1DM is approximately 9 ~ 10 years (104), and onset of T1DM occurs after six months of age. Accordingly, diabetes that develops before six months of age is classified as neonatal diabetes, in which other pathophysiologies, such as congenital malfunction of β-cells, are involved (105). However, several studies have revealed that T1DM associated with IEI frequently develops before six months of age (43, 103). In such patients, T1DM would be the first clinical symptom, and subsequently, immunodeficiency becomes clinically obvious. Accordingly, for T1DM patients whose onset is before six months of age, intensive genetic analysis, including IEI genes, is required.

Some IEI due to DNA repair defects, such as AT and Bloom syndrome, are reported to be associated with DM (106). In such IEI, insulin synthesis is generally maintained, and extremely increased insulin resistance has been suggested to contribute to the development of DM. In AT, lipodystrophy has been suggested as a cause of DM (52), and possibly, in Bloom syndrome, similar mechanisms would be involved, although precise analyses have not yet been reported.

A recent prospective analysis of 39 patients with AT showed a significant increase in HbA1c and fasting glucose levels with age (107). OGTT has good sensitivity for insulin resistance, and 7/39 (17.9%) patients had diabetes. In AT, metformin did not always lead to sufficient glycemic control (107).

## Endocrine and Metabolic Disorders Caused by HCT for IEI

### Impaired Reproductive Function Caused by HCT

Most IEI are due to defects in genes that are essential for hematopoietic cells to function normally, and supportive therapies, such as immunoglobulin G (IgG) infusions and antibiotics, are occasionally unsatisfactory for long-term outcomes. The only curative approach is the replacement of impaired cells by healthy donor hematopoietic stem cells and allogeneic HCT (allo HCT) (108–110). One of the clinical implications of HCT is the long-term complications that cause chronic and irreversible damage to various organs (111), including the endocrine glands. The major potential causes for long-term complications are conditioning regimens with cytotoxic agents and therapies for GVHD, including glucocorticoids (71).

The purpose of conditioning therapies is to ablate immune cells to allow stable engraftment of donor-derived HSCs. Classically, antineoplastic alkylating agents are used as key drugs in conditioning regimens for HCT. Although recent technical advances in HCT have enabled the reduction of the intensity of the regimen, alkylating agents are still widely used as the central drug (112, 113).

One of the common adverse effects of alkylating agents is germ cell toxicity. The clinical phenotypes of germ cell defects differ between female and male patients. In the ovaries, follicular structures, including oocytes, are essential for the differentiation and maintenance of steroidogenic ovarian cells, granulosa cells, and theca cells. Oocyte depletion due to alkylating agents causes hypogonadism with loss of estrogen and progesterone. In contrast, Leydig cells, which are steroidogenic cells in the testes, are viable in the absence of germ cells. Accordingly, normal androgen synthesis does not indicate the viability of germ cells in the testes, i.e., even after developing secondary sexual characteristics, future fertility of the male subjects is not warranted in IEI patients after HCT.

In contrast to malignant diseases, the conditioning regimen for IEI is generally less toxic, and the complications of HCT for patients with IEI would be different from that of malignant diseases, requiring careful evaluation of the toxicity of alkylating agents. However, evaluating the adverse effects of alkylating agents is not straightforward in IEI. First, the majority of severe IEI cases requiring HCT are male patients because of X-linked genetic diseases. Second, in contrast to hypogonadism, screening for isolated infertility in male patients is difficult, especially during childhood.

A recent report suggested that lower doses of alkylating agents for a reduced intensity conditioning regimen (RIC) would reduce the toxicity, including hypogonadism. A long-term follow-up study of 43 pediatric patients with non-malignant disease after HCT conditioned by RIC regimen revealed that all three postpubertal female patients before HCT resumed normal menstrual cycles post-HCT, and apparent hypogonadism were observed only one (5%) patient (112). A similar outcome was obtained from another cohort conditioned by fludarabine and melphalan based RIC (114). On the other hand, another report suggested that even RIC regimen would be a risk for hypogonadism in female patients (71). Treosulfan is an alkylating agent and increasingly used in recent pediatric practice because of less systemic toxicity than busulphan. By assessing bio markers for potential fertility, such as Anti-Müllerian hormone (AMH) and inhibin B, a treosulfan-based regimen confers a more favorable outcomes for gonadal reserve than fludarabine and melphalan or busulphan/cyclophosphamide based in both sexes (115).

With regard to the gonadal toxicity of alkylating agents, we should be aware that there is no uniformly “safe” or “toxic” alkylating agent dose threshold. Despite a cohort based on malignant diseases in which higher doses of alkylating agents are generally administered, a study of 214 cancer survivors found that despite a negative correlation between cyclophosphamide equivalent dose and sperm concentration, a firm threshold dose could not be established because azoospermia was observed even at the lowest dose (116).

Another issue for reproduction in IEI patients is age at HCT. Most patients with IEI receive HCT before the reproductive age (108, 112), resulting in limited options for the preservation of fertility before HCT, such as cryopreservation of gonadal tissues. However, at present, cryopreservation of gonadal tissues is considered an experimental technique, and this procedure may only be utilized for carefully selected patients as an experimental protocol (117). Further accumulation of these cases is necessary to elucidate the germ cell toxicity of RIC.

### Endocrine and Metabolic Disorders Caused by Prolonged Glucocorticoid Therapy

Long-term administration of glucocorticoids for GVHD after HCT or autoimmune response can cause various clinical problems, such as iatrogenic Cushing’s syndrome. The major adverse effects in the metabolic and endocrine systems include glucose intolerance, truncal obesity, and osteoporosis. For these adverse effects, careful and regular monitoring is required (118). Although the clinical presentation of iatrogenic Cushing’s syndrome in the pediatric population is similar to that in adults, there are some child-specific features, such as growth retardation. For the management of pediatric patients treated with systemic glucocorticoid therapy, frequent evaluation of linear growth and body weight change by plotting a growth chart is essential (119).

In addition to Cushing’s syndrome, patients treated with glucocorticoids are at high risk for adrenal insufficiency. Anti-inflammatory glucocorticoid treatment for IEI suppresses ACTH and CRH production from the pituitary gland and hypothalamus, respectively, decreasing endogenous cortisol synthesis (118). Subsequently, prolonged ACTH suppression leads to adrenal gland atrophy. Atrophic adrenal glands are not capable of promptly and properly synthesizing sufficient endogenous cortisol by abrupt cessation or rapid withdrawal of glucocorticoids, resulting in symptoms of adrenal insufficiency (118).

Furthermore, patients treated with low to medium doses of glucocorticoids are also at high risk for potentially life-threatening acute adrenal insufficiency. The low to medium dose of glucocorticoids suppresses the HPA axis, leading to adrenal atrophy. However, such a dose of glucocorticoid might not sufficiently tolerate physical stresses, such as fever, major injuries, seizure, and frequent vomiting, in which the required volume of glucocorticoid is dramatically increased (118). Physicians should always pay attention to adrenal insufficiency in patients treated with glucocorticoids.

## Conclusion remarks

In summary, we comprehensively reviewed and discussed the current understanding on the clinical features and the pathophysiology of endocrine disorders in IEI. We discussed the two major pathophysiology of endocrinopathy in IEI, autoimmune response and direct effects of the responsible genes, the details of each endocrinopathy, and potential endocrinopathy due to hematopoietic stem cell transplantation. Our expanding understanding on the molecular mechanisms of IEI have shed light on the new aspects of pathophysiology of endocrinopathies. Greater appreciation of these issues will provide valuable insights of the mechanisms underpinning endocrinopathies.

## Author Contributions

All authors conceptualized, planned, wrote, and approved the manuscript.

## Funding

The manuscript is partially funded by a Japanese nonprofit organization (NPO), Heart-link working project.

## Conflict of Interest

The authors declare that the research was conducted in the absence of any commercial or financial relationships that could be construed as a potential conflict of interest.

## Publisher’s Note

All claims expressed in this article are solely those of the authors and do not necessarily represent those of their affiliated organizations, or those of the publisher, the editors and the reviewers. Any product that may be evaluated in this article, or claim that may be made by its manufacturer, is not guaranteed or endorsed by the publisher.
